# Anti-Allergic Drug Suppressed Pancreatic Carcinogenesis via Down-Regulation of Cellular Proliferation

**DOI:** 10.3390/ijms22147444

**Published:** 2021-07-12

**Authors:** Kenta Kachi, Hiroyuki Kato, Aya Naiki-Ito, Masayuki Komura, Aya Nagano-Matsuo, Itaru Naitoh, Kazuki Hayashi, Hiromi Kataoka, Shingo Inaguma, Satoru Takahashi

**Affiliations:** 1 Department of Gastroenterology and Metabolism, Nagoya City University Graduate School of Medical Sciences, Nagoya 467-8601, Japan; k.kachi@med.nagoya-cu.ac.jp (K.K.); inaito@med.nagoya-cu.ac.jp (I.N.); khayashi@med.nagoya-cu.ac.jp (K.H.); hkataoka@med.nagoya-cu.ac.jp (H.K.); 2 Department of Experimental Pathology and Tumor Biology, Nagoya City University Graduate School of Medical Sciences, Nagoya 467-8601, Japan; h.kato@med.nagoya-cu.ac.jp (H.K.); ayaito@med.nagoya-cu.ac.jp (A.N.-I.); komura@med.nagoya-cu.ac.jp (M.K.); aya.ngn@med.nagoya-cu.ac.jp (A.N.-M.); 3 Department of Pathology, Nagoya City University East Medical Center, Nagoya 464-8547, Japan

**Keywords:** pancreatic cancer, montelukast, chemopreventive effect, drug repositioning, cysteinyl leukotriene receptor 1, leukotriene D_4_

## Abstract

Pancreatic cancer is a fatal disease, and thus its chemoprevention is an important issue. Based on the recent report that patients with allergic diseases have a low risk for pancreatic cancer, we examined the potential chemopreventive effect of anti-allergic agents using a hamster pancreatic carcinogenesis model. Among the three anti-allergic drugs administered, montelukast showed a tendency to suppress the incidence of pancreatic cancer. Further animal study revealed a significantly decreased incidence of pancreatic cancer in the high-dose montelukast group compared with controls. The development of the pancreatic intraepithelial neoplasia lesions was also significantly suppressed. The Ki-67 labeling index was significantly lower in pancreatic carcinomas in the high-dose montelukast group than in controls. In vitro experiments revealed that montelukast suppressed proliferation of pancreatic cancer cells in a dose-dependent manner with decreased expression of phospho-ERK1/2. Montelukast induced G1 phase arrest. Conversely, leukotriene D_4_ (LTD_4_), an agonist of CYSLTR1, increased cellular proliferation of pancreatic cancer cells with an accumulation of phospho-ERK1/2. In our cohort, pancreatic ductal adenocarcinoma patients with high CYSLTR1 expression showed a significantly unfavorable clinical outcome compared with those with low expression. Our results indicate that montelukast exerts a chemopreventive effect on pancreatic cancer via the LTD_4_–CYSLTR1 axis and has potential for treatment of pancreatic carcinogenesis.

## 1. Introduction

Pancreatic cancer is one of the most highly lethal cancers, with a 5-year survival rate of less than 10% [1]. Pancreatic ductal adenocarcinoma (PDAC), a major histological subtype of pancreatic cancer, exhibits highly malignant characteristics, and the current surgical therapy and chemotherapy strategies are not very effective for PDAC patients. Therefore, better understanding of the molecular characteristics of this cancer is required to develop a new strategy for the prevention of PDAC.

The field of drug repurposing (DR), the investigation of existing drugs for new therapeutic purposes, is one of the promising research avenues with potential to identify novel preventive methods or therapeutics. DR research can provide effective drugs in a short period of time and at a low cost because the drugs are compounds that have already passed clinical trials and have been demonstrated to have no problems with safety or pharmacokinetics.

A recent epidemiological survey revealed that the incidence of pancreatic cancer was significantly lower in patients with allergic diseases, such as asthma or nasal allergies, than allergy-free patients [2]. In addition, patients with more than five years of allergic medication history had a lower incidence of pancreatic cancer compared with those with a shorter history [2]. Another study reported that the use of a cysteinyl leukotriene receptor antagonist in asthma patients decreased the risk of lung, breast, colorectal and liver cancer in a dose-dependent manner [3]. However, the chemopreventive effects of anti-allergic drugs on pancreatic cancer development have not been fully elucidated.

In the present study, we hypothesized that anti-allergic drugs may exhibit preventative effects on pancreatic carcinogenesis and performed in vivo and in vitro experiments. We examined the effects of the anti-allergic drugs montelukast (a leukotriene receptor antagonist), bambuterol (a long-acting β-adrenoceptor agonist) and levocetirizine (a histamine H1 receptor antagonist) on the Syrian golden hamster model, in which *N*-nitrosobis (2-oxopropyl) amine (BOP) was used to induce pancreatic ductal carcinomas that are morphologically and molecularly similar to those in humans [4]. Our results suggest the possibility of using anti-allergic drugs to effectively and safely prevent pancreatic cancer development.

## 2. Results

### 2.1. Animal Experiments Demonstrated the Suppressive Effects of Montelukast on Pancreatic Carcinogenesis in BOP-Treated Hamsters

**Figure 1:**
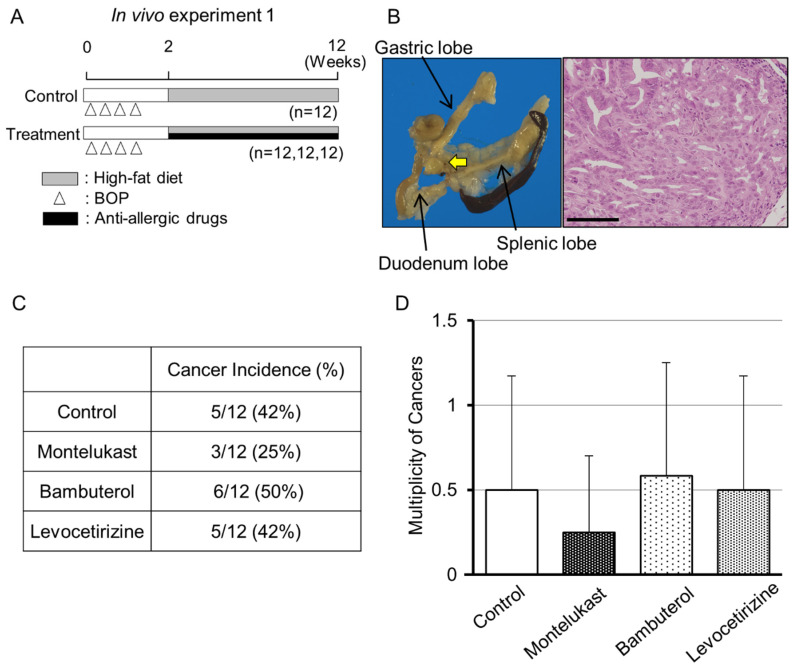
Montelukast exhibited a suppressive effect on pancreatic carcinogenesis in the hamster pancreatic cancer model. (**A**) Experimental design for the BOP-induced pancreatic cancer model in hamster. Anti-allergic drugs: montelukast, bambuterol and levocetirizine. (**B**) Representative images of pancreatic cancer in the hamster model (the cancer lesion is indicated by the yellow arrow) and H&E staining of the cancer lesion. Bar = 100 µm. (**C**) Cancer incidence and (**D**) multiplicity in the four groups.

To examine the anti-carcinogenic effects of anti-allergic drugs, montelukast, bambuterol and levocetirizine were administered to the BOP-induced pancreatic cancer model of hamsters. The design of in vivo experiment 1 is shown in Figure 1A. All animals remained healthy throughout the experimental period. At the end of the study, only the levocetirizine group had a higher body weight than the control group (Supplementary Table S1A). Representative images of pancreatic lesions are shown in Figure 1B. The body and organ weights, complete blood count and biochemical tests of all groups are shown in Supplementary Figure S1 and Table S1.

Among the three drugs administered in the hamster pancreatic model, only montelukast showed a tendency to suppress the number of pancreatic cancer lesions in the animal model (Figure 1C,D and Supplementary Table S2).

### 2.2. High-Dose Montelukast Significantly Inhibited Pancreatic Cancer Development by Suppressing Cellular Proliferation

**Figure 2:**
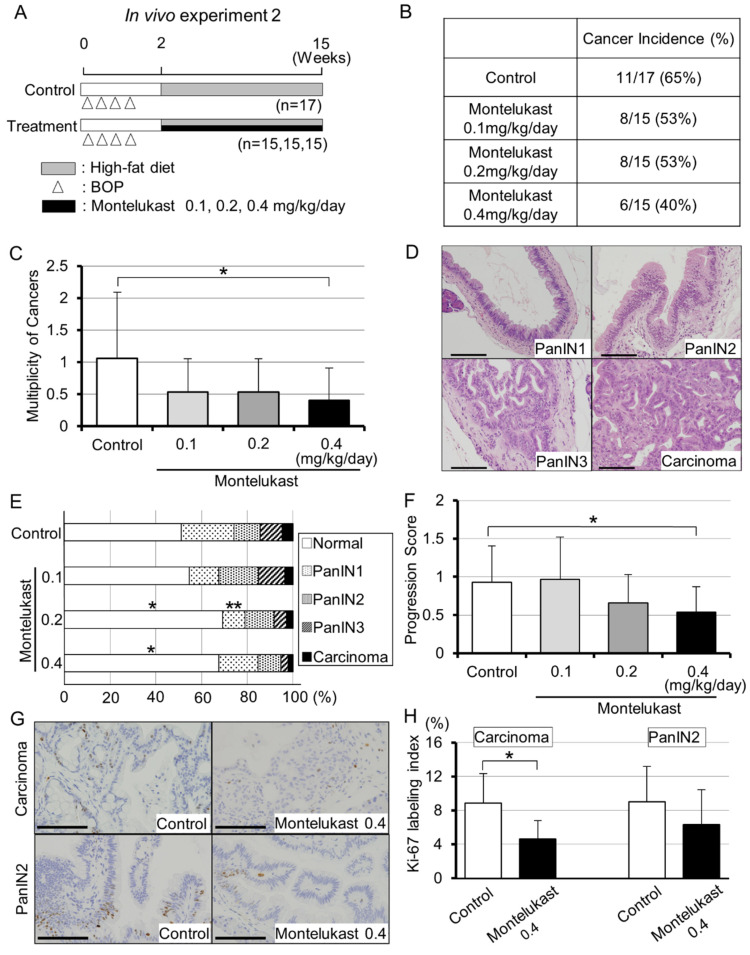
High-dose montelukast significantly suppressed pancreatic carcinogenesis and inhibited cancer development by suppressing cellular proliferation in the hamster model. (**A**) Experimental design for the BOP-induced pancreatic cancer model in hamster. (**B**) Cancer incidence and (**C**) multiplicity in the four groups (control, low, medium and high dose of montelukast). (**D**) H&E staining of PanIN and carcinoma lesions in hamster pancreases. Bars = 100 µm. (**E**) The proportion of normal, PanINs and carcinoma in all pancreatic ducts (diameter > 200 µm) of duodenal lobes and (**F**) progression score calculated by weighting respective lesions. (**G**) Representative immunohistochemical staining for Ki-67 and (**H**) Ki-67 labeling index in carcinoma and PanIN2 lesion of the control and montelukast high-dose group. Bars = 100 µm. * *p* < 0.05 and ** *p* < 0.01 compared with controls.

Several reports have uncovered the anti-cancer effects of montelukast [5,6,7,8,9,10]. Therefore, we focused on the suppressive effects of montelukast and performed further investigations. The design of in vivo experiment 2 is shown in Figure 2A. There was no significant difference in the mean body, liver and kidney weight among the groups (Supplementary Figure S2 and Supplementary Table S3). Cancer incidence at each montelukast concentration is shown in Figure 2B. The multiplicity of cancers was significantly decreased in the high-dose montelukast group (0.4 mg/kg/day) compared with the control group (Figure 2C and Supplementary Table S4). Representative images of the pancreatic lesions are shown in Figure 2D. The progression of pancreatic intraepithelial neoplasia (PanIN) lesions was also significantly suppressed in the high-dose montelukast group compared with the control group (Figure 2E,F and Supplementary Table S5). The Ki-67 labeling index was significantly lower in the pancreatic carcinomas in the high-dose montelukast group than those in the control group (Figure 2G,H).

### 2.3. CYSLTR1 Regulated the Cellular Proliferation of PDAC Cells

**Figure 3:**
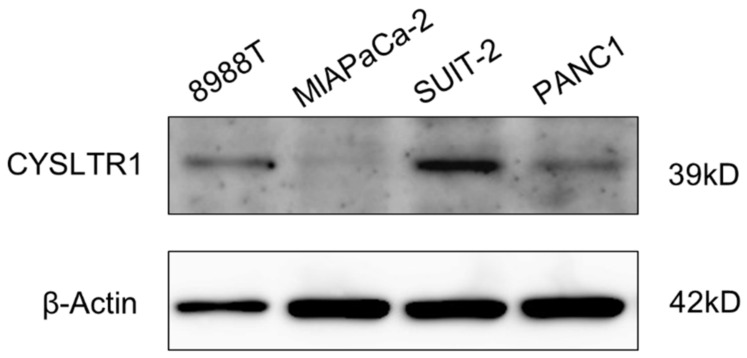
CYSLTR1 expression in 8988T, MIAPaCa-2, SUIT-2 and PANC1 cells.

**Figure 4:**
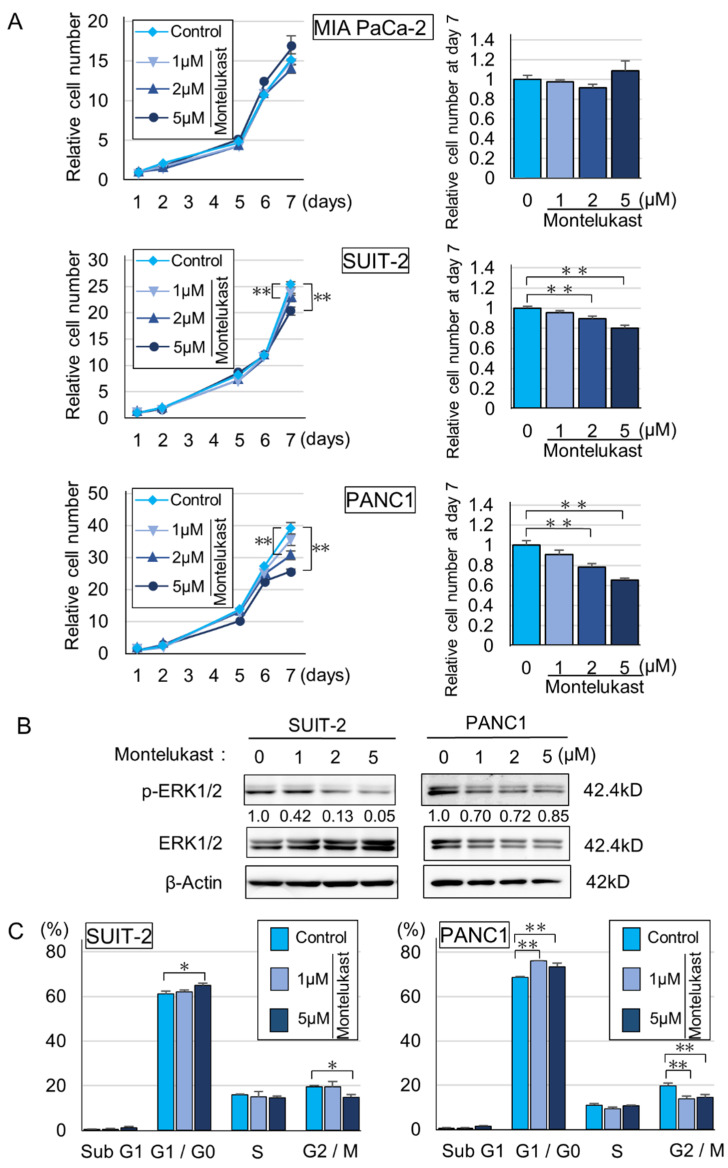
Montelukast suppressed cellular proliferation of PDAC cells with down-regulation of p-ERK. (**A**) Graphs of relative cell number compared with day 1 (left) and bar graphs of relative cell numbers compared with the control at day 7 (right) quantified by CellTiter 96 in the low-CYSLTR1-expressing cell line (MIAPaCa-2) and high-CYSLTR1-expressing cell lines (SUIT-2 and PANC1). PDAC cells were treated with montelukast at day 1. Data are presented as mean ± SD, *n* = 3 per group, ** *p* < 0.01 compared with controls. (**B**) Immunoblot assay of p-ERK in high CYSLTR1-expressing cells (SUIT-2 and PANC1) treated with montelukast for 6 days. (**C**) Cell cycle analyses of SUIT-2 and PANC1. Data are presented as means ± SD. Experiments were performed in triplicate. * *p* < 0.05 and ** *p* < 0.01 compared with controls.

**Figure 5:**
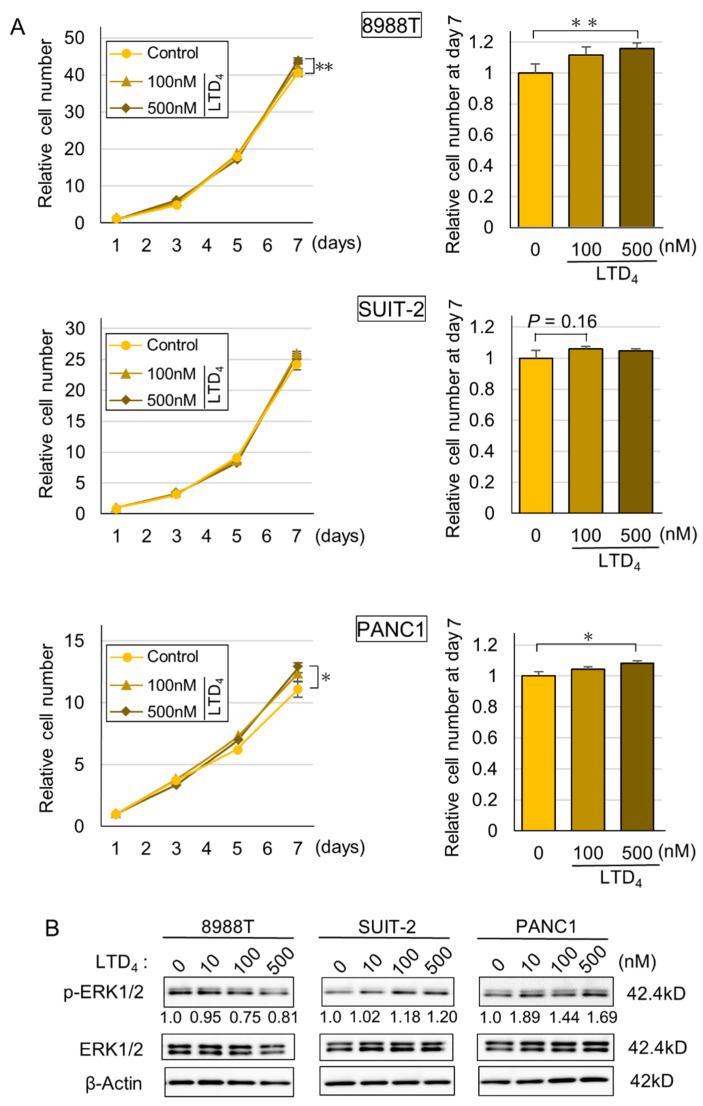
LTD_4_ increased cellular proliferation of PDAC cells with an accumulation of p-ERK. (**A**) Graphs of relative cell number compared with day 1 (left) and bar graphs of relative cell numbers compared with controls at day 7 (right) quantified by CellTiter 96 in high-CYSLTR1-expressing cells (8988T, SUIT-2 and PANC1). PDAC cells were treated with LTD_4_ at day 1. Data are presented as mean ± SD, *n* = 3 per group, * *p* < 0.05 and ** *p* < 0.01 compared with controls. (**B**) Immunoblot of p-ERK in high-CYSLTR1-expressing cells treated with LTD_4_ for 24 h.

To uncover the suppressive effects of montelukast on pancreatic carcinogenesis, we performed in vitro experiments. Immunoblot assays showed that cysteinyl leukotriene receptor 1 (CYSLTR1), a receptor that montelukast antagonizes, was expressed in 8988T, SUIT-2 and PANC1 cell lines. In contrast, MIAPaCa-2 cells expressed CYSLTR1 at nearly undetectable levels (Figure 3 and Supplementary Figure S3). Montelukast significantly suppressed the cellular proliferation of SUIT-2 and PANC1, which express CYSLTR1 (Figure 3 and Figure 4A). Down-regulation of p-ERK, which is a key protein in the ERK1/2 pathway in the down-stream of CYSLTR1 signaling, was detected (Figure 4B and Supplementary Figure S4). Cell cycle and immunoblot analyses revealed that montelukast induced G0/G1 arrest without inducing apoptosis in PDAC cells (Figure 4C and Supplementary Figures S5 and S6). In contrast, leukotriene D_4_ (LTD_4_), an agonist of CYSLTR1, accelerated cellular proliferation of PDAC cells with an accumulation of p-ERK (Figure 5A,B and Supplementary Figure S7).

**Figure 6:**
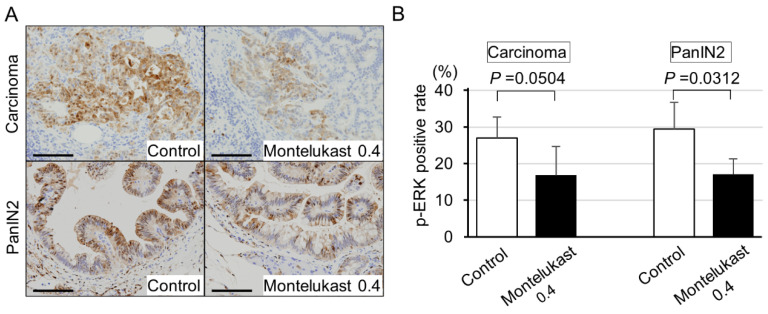
Montelukast suppressed p-ERK expression in pancreatic lesions. (**A**) Representative images for p-ERK immunostaining. (**B**) p-ERK positive rate in carcinoma and PanIN2 lesions with or without montelukast treatment. Bars = 100 µm.

Based on our observations that montelukast suppressed p-ERK accumulation in human PDAC cells, we performed immunohistochemical analyses for p-ERK expression in hamster pancreatic lesions. The positive rate for p-ERK tended to be lower in the high-dose montelukast group than those in the control group (Figure 6A,B).

### 2.4. High CYSLTR1 Expression Was Associated with Poor Prognosis in PDAC Patients

**Figure 7:**
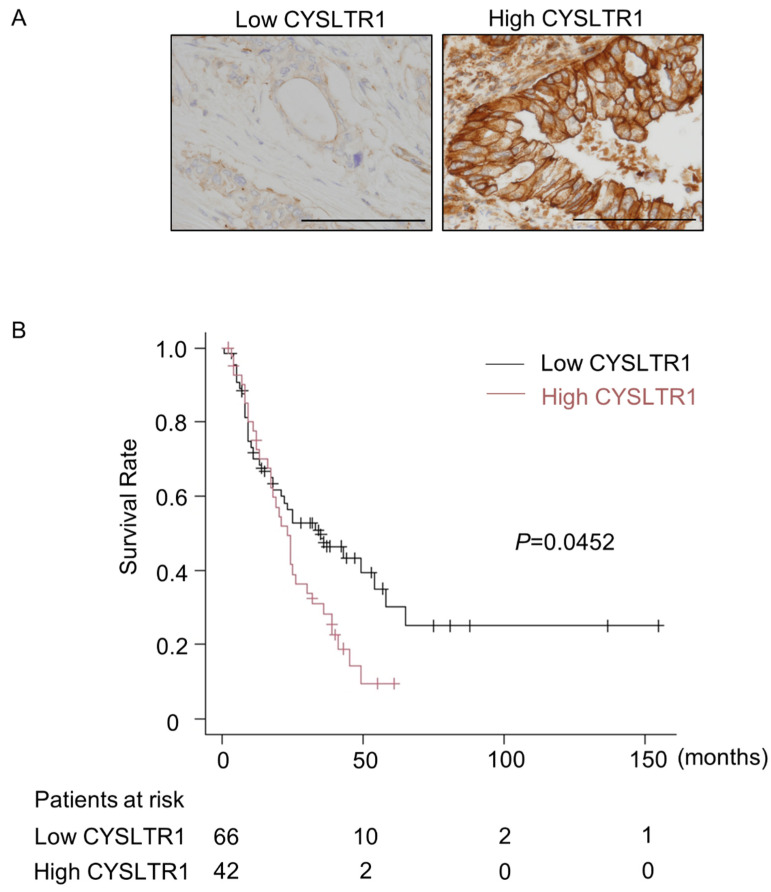
High CYSLTR1 expression is associated with poor prognosis in PDAC patients. (**A**) Representative images of CYSLTR1 immunohistochemistry in human PDAC. Images of the low-CYSLTR1-expressing group and the high-CYSLTR1-expressing group are shown. Bars = 100 µm. (**B**) Kaplan–Meier overall survival curve in PDAC patients with high CYSLTR1 expression and low CYSLTR1 expression (*n* = 108). *p*-value was calculated by log-rank test.

Finally, we performed immunohistochemical analyses for CYSLTR1 expression in PDAC tissue arrays. Representative images for CYSLTR1 immunohistochemistry in human PDAC specimens are shown in Figure 7A. The Cohen’s kappa coefficient was 0.69 (95%CI: 0.55–0.82). Clinicopathological features according to CYSLTR1 expression are summarized in Supplementary Table S6. PDAC patients with high CYSLTR1 expression had a significantly worse clinical outcome than those with low CYSLTR1 expression (Figure 7B).

## 3. Discussion

The prognosis of pancreatic cancer has remained poor despite the improvement in treatment modalities and therapeutics. Therefore, a new preventive strategy is required based on a better understanding of the molecular characteristics of this cancer. In the present study, we conducted DR experiments to find a drug with a chemopreventive effect on pancreatic cancer. Our results demonstrate the inhibitory effects of montelukast on pancreatic carcinogenesis through the inhibition of cellular proliferation.

DR is a strategy for finding new treatments among existing approved medicines. In recent years, the number of reports on DR has increased [11]. Administration of angiotensin II receptor blockers might improve clinical outcomes of patients with prostate cancer or pancreatic cancer [12,13]. Regarding anti-allergic drugs, studies reported that breast cancer patients taking H1 antihistamines had better survival rates compared with those not taking H1 antihistamines [14,15]. Since the development of oncology drugs is a lengthy and costly process with high attrition rates, DR represents a powerful alternative strategy for the identification of effective treatments [16]. The benefits of DR are as follows: availability of pharmacokinetics, pharmacodynamics and posology data; knowledge of safety and toxicity, including rare adverse events; clinical experience derived from the original indications; widespread availability; low cost; and understanding of mechanisms of action and molecular targets [17]. Our findings suggest that montelukast may be applied as a repositioned drug for pancreatic cancer.

Leukotrienes have been reported to be involved in cancer development, metastases and cachexia [18]. LTD_4_, one of the most potent leukotrienes, is synthesized primarily by stimulated leukocytes, and the effects of LTD_4_ are mainly mediated thorough CYSLTR1, which is a high-affinity receptor for LTD_4_ [19]. The LTD_4_–CYSLTR1 axis has been reported to promote proliferation and induce transcriptional activity of potentially oncogenic genes [3,19]. Increased expression of CYSLTR1 has been reported in colorectal, gastric and breast cancers, and its high expression is associated with decreased survival in these cancer patients [3,20,21,22]. Montelukast, an antagonist against CYSLTR1, inhibited cell proliferation of HCT-116 cells (a human colon cancer cell line) in a dose-dependent manner [23]. These observations are consistent with our findings in this study that demonstrate that montelukast prevents pancreatic carcinogenesis.

In the present study, we uncovered the anti-carcinogenic effects of montelukast in a hamster pancreatic carcinogenesis model. The effects of montelukast on pancreatic carcinogenesis have never been reported. Our results were also the first to describe the risk of high CYSLTR1 expression in PDAC on patient survival. The anti-carcinogenic effects of montelukast are likely from the anti-proliferation effects of montelukast on pancreatic neoplastic cells. This observation was similar to the results in the Lewis lung carcinoma model, in which Ki-67 expression in tumor specimens was significantly reduced in the montelukast group compared with the control group [5]. In our in vitro experiments, the proliferation of PDAC cells was suppressed by montelukast with reduced expression of p-ERK1/2. In addition, cell cycle analyses showed that montelukast caused G1 arrest in PDAC cells without inducing apoptosis.

Several reports have investigated the suppression of cancer cell growth by montelukast, and in most of the reports, the montelukast concentrations that induced suppressive effects were extremely high (12.5–100 µM) [5,6,7,8,9,10]. In contrast, in the present study, we observed significant inhibitory effects of cellular proliferation at 2 to 5 µM of montelukast. When an adult human is given the recommended dose of montelukast (10 mg once daily), the maximum plasma concentration is approximately 1 µM. Therefore, the concentration used in our study is practical considering its application to humans. We also tried to clarify the pathways that act on PDAC cells through stromal cells, but the results were equivocal (data not shown). We additionally performed microarray analysis on RNA from hamster pancreatic tissue and found that Smad3 showed a significant decrease in expression in response to montelukast treatment of hamsters, but we could not confirm the decrease in expression in in vitro experiments (data not shown). We found that LTD_4_ treatment of pancreatic cancer cell lines with high expression of CYSLTR1 led to cellular proliferation with ERK1/2 phosphorylation. These results indicate that montelukast at safe concentrations may inhibit pancreatic carcinogenesis through suppression of the LTD_4_–CYSLTR1–ERK axis.

Dysregulation of signaling pathways that affect cell survival and proliferation is a major driver in carcinogenesis [5]. Mitogen-activated protein kinase (MAPK) cascades, especially those involving ERK1/2 activated by MAPK/ERK kinase (MEK) 1/2 dual-specificity protein kinases, promote the survival and migration of cancer cells [5,24,25]. In the present study, our results show that montelukast may inhibit pancreatic carcinogenesis through the suppression of the LTD_4_–CYSLTR1–ERK axis. Our observations are in line with previous reports showing that LTD_4_–CYSLTR1 signaling regulates cellular proliferation in human colon cancer and intestinal epithelial cells [26,27]. Furthermore, montelukast-induced cell death with decreased ERK1/2 phosphorylation was reported in human lung cancer cell lines A549 and CL1-5 [5]. However, in our experiments, montelukast did not induce apoptotic cell death in PDAC cells based on the practical concentration of montelukast. In addition, montelukast-induced reduction in migration with decreased ERK1/2 phosphorylation was reported in the endothelial cell line EA.hy926 [28], which might correlate with the anti-carcinogenic effects of montelukast by inhibiting angiogenesis [26]. Thus, montelukast may be used as an anti-carcinogenic drug that suppresses several critical pathways indispensable for carcinogenesis.

## 4. Materials and Methods

### 4.1. Animals, Diet and Chemicals

Five-week-old female Syrian golden hamsters weighing approximately 80 g were purchased from Japan SLC, Inc. (Shizuoka, Japan). The hamsters were kept in cages on hardwood chip bedding (*n* = 3 hamsters/cage) and kept at 22 ± 2 °C and 55 ± 5% humidity. The animals were maintained under specific pathogen-free conditions with a 12 h light/dark cycle. The Quick Fat diet (crude fat, 13.6%; crude protein, 24.2%; total calories, 4.06 kcal/g) (CLEA Japan, Tokyo, Japan) was used as a high-fat regimen. Montelukast, bambuterol and levocetirizine were purchased from Tokyo Chemical Industry Co., Ltd. (Tokyo, Japan). BOP was obtained from Toronto Research Chemicals Inc. (Toronto, ON, Canada).

### 4.2. Animal Experiments

All animal experiments were performed using protocols approved by the Institutional Animal Care and Use Committee of Nagoya City University Graduate School of Medical Sciences (No. H28M-14, approved on 20 April 2016 and H29M-55, approved on 13 November 2017). Animal experiments were performed according to the previous reports [29,30].

In experiment 1 (Figure 1A), 48 female hamsters at 6 weeks of age were given four subcutaneous injections of BOP (on days 1, 3, 5 and 7) at a dose of 10 mg/kg body weight. One week after the last injection of BOP, the animals were randomized into four groups, and all groups were fed a high-fat diet. Animals were given drinking water containing 0.2 mg/kg/day montelukast, 0.1 mg/kg/day levocetirizine or 0.2 mg/kg/day bambuterol for 10 weeks; the control group was given water without any drugs. The drug doses were similar to those used in humans. The experiment was terminated at 12 weeks after the first injection. All hamsters were sacrificed under anesthesia, and the pancreas, liver, lungs and kidneys were removed.

In experiment 2 (Figure 2A), 62 female hamsters (6 weeks of age) were given four subcutaneous injections of BOP as described above, randomized into four groups and fed a high-fat diet. The montelukast low-dose group, medium-dose group and high-dose group were given drinking water containing 0.1, 0.2 and 0.4 mg/kg/day for 13 weeks, respectively (0.2 mg/kg/day corresponds to the usual dose for humans); the control group was given water without drugs. At 15 weeks after the first injection, the animals were sacrificed to remove organs and three anatomical parts of the pancreas (gastric, splenic and duodenal lobes).

### 4.3. Histopathological Examination

Organs were fixed in 10% phosphate-buffered formalin. The tissues were routinely processed, embedded in paraffin, serialized to a thickness of 4 µm and stained with hematoxylin and eosin (H&E) to evaluate the histopathological features. All pancreas lobes were cut on the minor axis every 2 mm, and all parts were analyzed. Lesions of all pancreatic ducts (diameter > 200 µm) in duodenal lobes were classified as normal, PanIN1, PanIN2, PanIN3 or carcinoma. The multiplicity of cancer is the average number of cancer foci that occurred in animals for each group. The progression score was calculated by weighting respective lesions (normal = 0, PanIN1 = 1, PanIN2 = 2, PanIN3 = 3, carcinoma = 4).

### 4.4. Immunohistochemistry

Immunohistochemistry was performed using a Leica Bond-Max (Leica Biosystems, Bannockburn, IL, USA) with a Leica Refine detection kit (Leica Biosystems). Antibodies and conditions for immunohistochemistry are shown in Supplementary Table S7.

### 4.5. Cell Culture

Human pancreatic cancer cell lines PA-TU-8988T, MIA PaCa-2, SUIT-2 and PANC1 were obtained from the American Type Culture Collection (ATCC, Rockville, MD, USA). Cells were maintained in RPMI1640 (Wako Pure Chemical Industries Co. Ltd., Osaka, Japan) supplemented with 10% fetal bovine serum (FBS).

Cells were seeded in 12-well plates (5.0 × 10^4^ per well) with or without montelukast (1, 2, 5 µM) or leukotriene D_4_ (LTD_4_) (Cayman Chemicals, Ann Arbor, MI, USA) (10, 100, 500 nM). We took care to ensure that the montelukast concentrations were close to those of human plasma at normal administration. After incubation, cell numbers were measured using CellTiter 96^®^ Aqueous One Solution (Promega, Madison, WI, USA) according to the manufacturer’s protocol. Experiments were performed in triplicate.

### 4.6. Cell Cycle Analysis

SUIT-2 and PANC1 cells were treated with 1 or 5 µM montelukast for 4 days. Cell cycle analyses were performed by using the Guava Cell Cycle Reagent and Guava easyCyte Single system (Merck, Darmstadt, Germany) according to the manufacturer’s protocol.

### 4.7. Immunoblotting

Whole cell lysates were prepared and subjected to immunoblot analyses using a previously reported procedure [31,32]. Information on the antibodies used in the immunoblot assays is summarized in Supplementary Table S8. Signal intensity was measured by ImageJ software (National Institutes of Health, Bethesda, MD, USA).

### 4.8. Tissue Samples, Clinical Information and CYSLTR1 Expression in PDAC

This study was approved by the Institutional Review Board at Nagoya City University Graduate School of Medical Sciences and conformed to the guidelines of the Declaration of Helsinki. Formalin-fixed paraffin-embedded samples of 108 PDACs resected at the Nagoya City University Hospital from 2005 to 2018 were collected, along with patient clinical information (Supplementary Table S6). After surgery, patients were followed for up to 155 months. PDACs were divided into two groups according to CYSLTR1 expression: low CYSLTR1 (partial membrane expression with strong or weak staining, *n* = 65) and high CYSLTR1 (complete membrane expression with strong staining, *n* = 43). CYSLTR1 expression on the cytomembrane of tumor cells was independently evaluated by two researchers (K.K. and S.I.). In cases of discordant conclusions, the results were confirmed by discussion.

### 4.9. Statistical Analysis

Differences in the quantitative data between groups were compared by one-way ANOVA, Dunnett’s post hoc test, Student’s *t*-test and the χ2 test as appropriate using Graph Pad Prism 8 (GraphPad Software, Inc., La Jolla, CA, USA). Data are expressed as mean ± SD. Kaplan–Meier survival estimate with log-lank test was performed by EZR software version 1.54 [33].

## 5. Conclusions

Our findings suggest that montelukast suppressed pancreatic carcinogenesis by inhibiting the cellular proliferation of pancreatic cancer cells. Widely used anti-allergic drugs may be used as a chemopreventive treatment for pancreatic cancer.

## Data Availability

The data presented in this study are available upon request from the corresponding author.

## Supplementary Materials

The following are available online at https://www.mdpi.com/article/10.3390/ijms22147444/s1, Figure S1: Mean body weight of hamsters given anti-allergic agents in in vivo experiment 1, Figure S2: Mean body weight of hamsters given montelukast (control, 0.1, 0.2, 0.4 mg/kg/day) in in vivo experiment 2, Figures S3–S4: Whole Western blots (uncropped images) showing all bands with molecular weight markers, Figure S5: Cell cycle analysis of SUIT-2 and PANC1 treated with or without montelukast, Figure S6: Montelukast did not induce apoptosis in PDAC cells, Figure S7: Whole Western blots (uncropped images) showing all bands with molecular weight markers, Table S1: The body, liver, kidney and lung weights, complete blood count and biochemical test of the hamsters at the conclusion of in vivo experiment 1, Table S2: Total cancer number and multiplicity in in vivo experiment 1, Table S3: The body, liver and kidney weights of the hamsters at the conclusion of in vivo experiment 2, Table S4: Total cancer number and multiplicity in the four groups (control, low, medium and high dose of montelukast) in in vivo experiments 2, Table S5: The number of normal, PanINs and carcinomas in all pancreatic ducts (diameter > 200 µm) of duodenal lobes, and progression score in the four groups ((A) control, (B) low,(C) medium and (D) high dose of montelukast) in in vivo experiments 2, Table S6: Patient characteristics, Table S7: Antibodies and dilutions for immunohistochemistry, Table S8: Antibodies and dilutions for immunoblotting.
