# Supplementary material for: Anti-Allergic Drug Suppressed Pancreatic Carcinogenesis via Down-Regulation of Cellular Proliferation
Source: Int J Mol Sci. 2021 Jul 12;22(14):7444. doi: 10.3390/ijms22147444 (PMC8304964; doi:10.3390/ijms22147444)
Supplement: Supplementary file 1 [file ijms-22-07444-s001.zip › ijms-1273421-suppl-conversion.pdf]

International Journal of *Molecular Sciences*

MDPI

Supplementary Materials

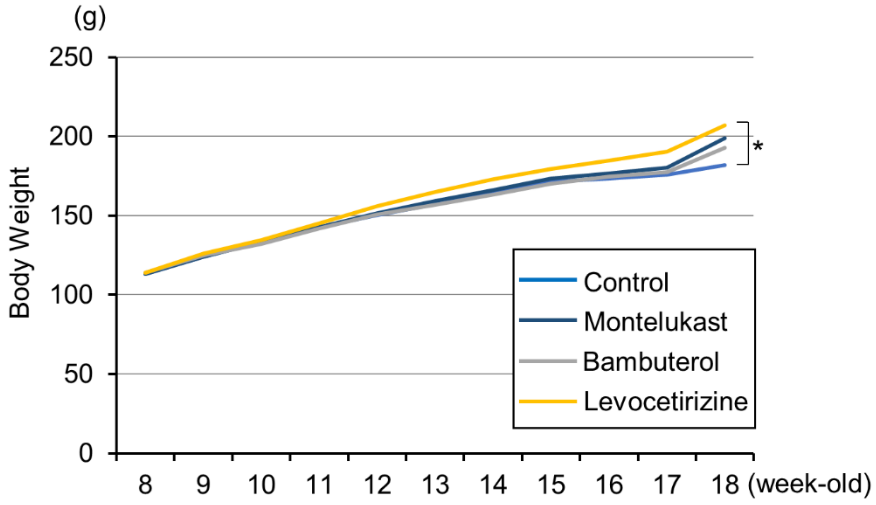

Figure S1. Mean body weight of hamsters given antiallergic agents in in vivo experiment 1. *$p < 0.05$ compared with controls.

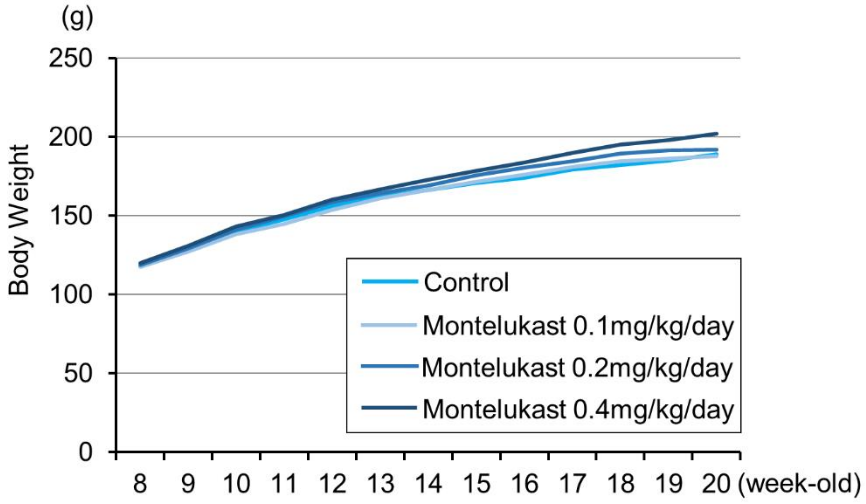

Figure S2. Mean body weight of hamsters given montelukast (control, 0.1, 0.2, 0.4 mg/kg/day) in in vivo experiment 2.

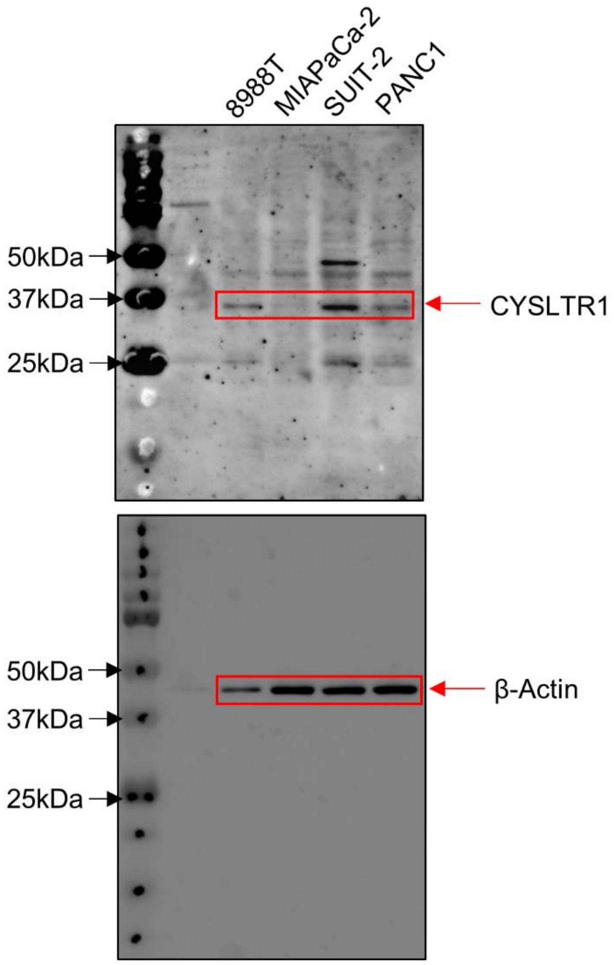

Figure S3. Whole western blots (uncropped images) showing all bands with molecular weight markers. These gels correspond to those shown in Figure 3.

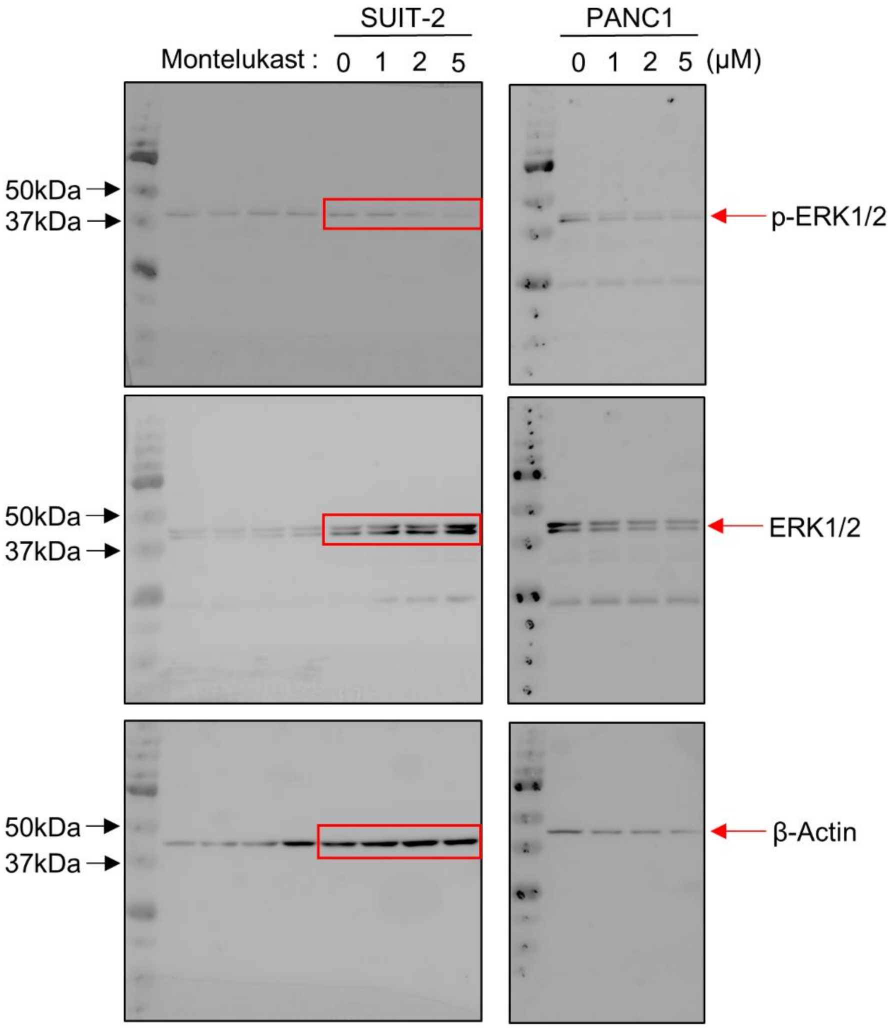

Figure S4. Whole western blots (uncropped images) showing all bands with molecular weight markers. These gels correspond to those shown in Figure 4B.

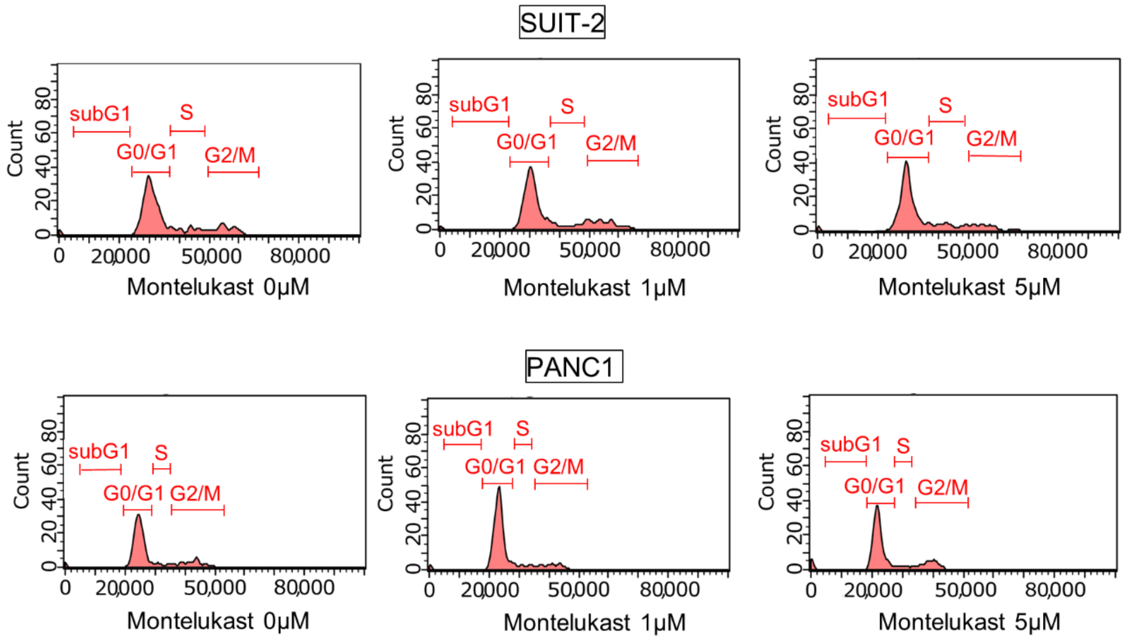

Figure S5. Cell cycle analysis of SUIT-2 and PANC1 treated with or without montelukast.

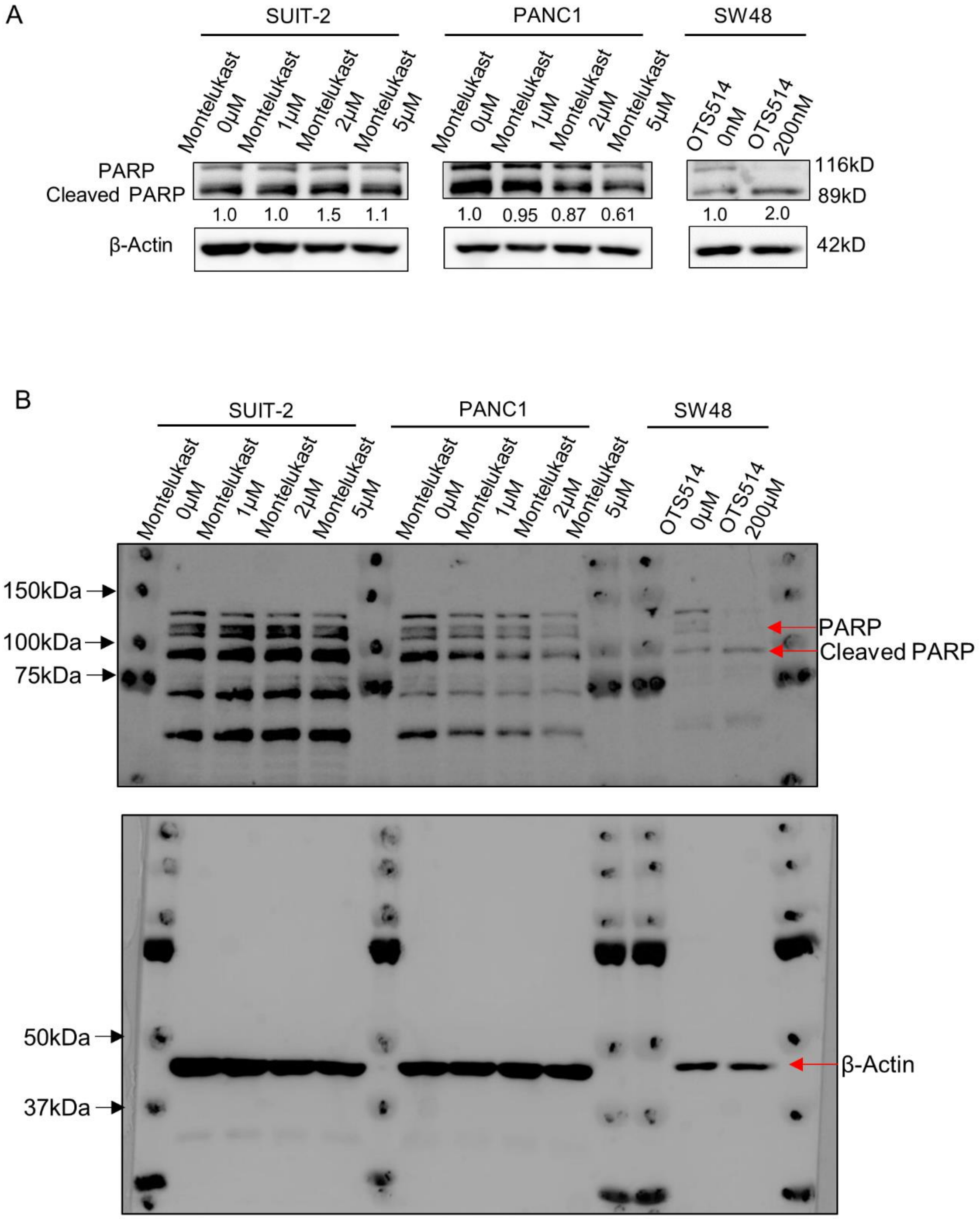

Figure S6. Montelukast did not induce apoptosis in PDAC cells. (**A**) Immunoblot analyses of PARP in high CYSLTR1-expressing cells (SUIT-2 and PANC1) treated with montelukast for 6 days. SW48 treated with OTS514 was used as a positive control. (**B**) Whole western blots (uncropped images) showing all bands with molecular weight markers. These gels correspond to those shown in (A).

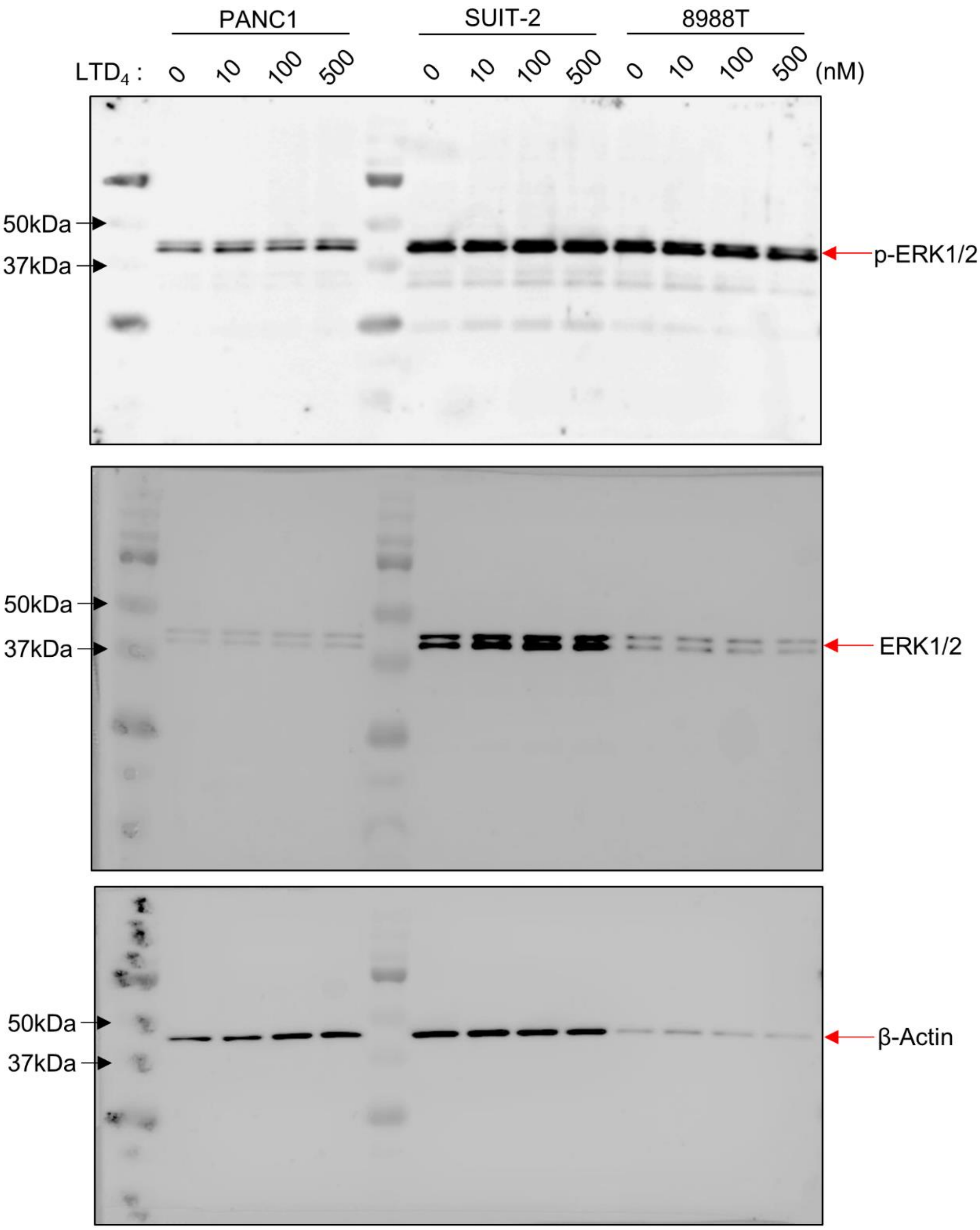

Figure S7. Whole western blots (uncropped images) showing all bands with molecular weight markers. These gels correspond to those shown in Figure 5B.

Table S1. (**A**) The body, liver, kidney and lung weights, (**B**) complete blood count and (**C**) biochemical test of the hamsters at the conclusion of in vivo experiment 1. Values are means ± SD, n = 12 in each group, *p < 0.05 compared with controls.

(A) The Body, Liver, Kidney and Lung Weights of Hamsters

Treatment	Animal Number	Body (g)	Liver (g)	Relative Liver (%)	Kidney(g)	Relative Kidney (%)	Lung (g)	Relative Lung (%)
Control	12	182 ± 18	10.5 ± 1.7	5.8 ± 0.7	1.5 ± 0.2	0.8 ± 0.08	1.0 ± 0.1	0.5 ± 0.04
Montelukast	12	199 ± 29	12.6 ± 2.0 *	5.5 ± 0.3	1.6 ± 0.1	0.8 ± 0.14	1.0 ± 0.1	0.5 ± 0.11
Bambuterol	12	193 ± 20	11.3 ± 1.9	5.6 ± 0.5	1.5 ± 0.2	0.8 ± 0.05	1.0 ± 0.1	0.5 ± 0.06
Levocetirizine	12	207 ± 22 *	12.6 ± 1.7 *	5.3 ± 0.7	1.6 ± 0.2	0.8 ± 0.04	1.0 ± 0.1	0.5 ± 0.05

(B) Complete Blood Count

Treatment	White Blood Cell (/μL)	Hemoglobin (g/dL)	Platelet (×10^4/μL)	Eosinophil (/μL)
Control	5743 ± 884	15.5 ± 0.8	19.2 ± 3.4	57.2 ± 15.6
Montelukast	6320 ± 1124	15.3 ± 0.7	22.5 ± 2.9	62.7 ± 25.3
Bambuterol	6900 ± 1653	15.6 ± 0.5	20.4 ± 4.2	45.5 ± 26.2
Levocetirizine	6282 ± 1005	15.8 ± 0.4	20.3 ± 2.1	52.5 ± 18.2

(C) Biochemical Test

Treatment	LDL Cholesterol (mg/dL)	HDL Cholesterol (mg/dL)	Total Cholesterol (mg/dL)	Triglyceride (mg/dL)	Amylase (U/L)
Control	97 ± 27	131 ± 26	262 ± 50	451 ± 128	4143 ± 1268
Montelukast	127 ± 40	137 ± 30	317 ± 52 *	604 ± 160 *	4627 ± 772
Bambuterol	119 ± 32	152 ± 23	316 ± 48 *	556 ± 161	4468 ± 624
Levocetirizine	114 ± 29	153 ± 17	309 ± 41	572 ± 116	4389 ± 733

*p < 0.05 compared with controls.

Table S2. Total cancer number and multiplicity in in vivo experiment 1. Data are presented as mean ± S.D., n = 12 per group.

Treatment	**Animal Number**	**Total Cancer Number**	**Multiplicity of Cancers**
Control	12	6	0.50 ± 0.67
Montelukast	12	3	0.25 ± 0.45
Bambuterol	12	7	0.58 ± 0.67
Levocetirizine	12	6	0.50 ± 0.67

Table S3. The body, liver and kidney weights of the hamsters at the conclusion of in vivo experiment 2.

**Treatment**	**Animal Number**	**Body (g)**	**Liver (g)**	**Relative Liver (%)**	**Kidney (g)**	**Relative Kidney (%)**
Control	17	190 ± 23	10.9 ± 1.9	5.7 ± 0.5	1.46 ± 0.12	0.77 ± 0.05
Montelukast 0.1 mg/kg/day	15	188 ± 25	10.4 ± 2.2	5.5 ± 0.6	1.42 ± 0.17	0.75 ± 0.06
Montelukast 0.2 mg/kg/day	15	193 ± 18	11.0 ± 1.5	5.7 ± 0.3	1.38 ± 0.10	0.72 ± 0.07
Montelukast 0.4 mg/kg/day	15	203 ± 20	11.9 ± 1.8	5.8 ± 0.4	1.46 ± 0.21	0.71 ± 0.05

Values are means ± SD, n = 17 (control), n = 15 (each montelukast-treated group).

Table S4. Total cancer number and multiplicity in the four groups (control, low, medium and high dose of montelukast) in in vivo experiments 2.

Treatment	Animal Number	Total Cancer Number	Multiplicity of Cancers
Control	17	18	1.06 ± 1.03
Montelukast 0.1mg/kg/day	15	8	0.53 ± 0.52
Montelukast 0.2mg/kg/day	15	8	0.53 ± 0.51
Montelukast 0.4mg/kg/day	15	8	0.40 ± 0.51 *

*$p < 0.05$ compared with controls. Data are presented as mean ± S.D., $n = 17$ (control), $n = 15$ (each montelukast-treated group).

Table S5. The number of normal, PanINs and carcinoma in all pancreatic ducts (diameter > 200 μm) of duodenal lobes, and progression score in the four groups ((**A**) control, (**B**) low, (**C**) medium and (**D**) high dose of montelukast) in in vivo experiments 2. *$p < 0.05$ and **$p < 0.01$ compared with controls.

(A)

Individual Animals	Normal Number	PanIN1 Number	PanIN2 Number	PanIN3 Number	Carcinoma Number	Progression Score
Control 1	5 (38.5%)	5 (38.5%)	0 (0%)	2 (15.4%)	1 (7.7%)	1.36
Control 2	5 (50%)	1 (10%)	2 (20%)	1 (10%)	1 (10%)	1.20
Control 3	1 (14.3%)	2 (28.6%)	2 (28.6%)	1 (14.3%)	1 (14.3%)	1.86
Control 4	2 (40%)	2 (40%)	1 (20%)	0 (0%)	0 (0%)	0.80
Control 5	9 (47.4%)	4 (21.1%)	2 (10.5%)	2 (10.5%)	2 (10.5%)	1.16
Control 6	2 (66.7%)	1 (33.3%)	0 (0%)	0 (0%)	0 (0%)	0.33
Control 7	3 (33.3%)	1 (11.1%)	3 (33.3%)	2 (22.2%)	0 (0%)	1.44
Control 8	5 (45.5%)	4 (36.4%)	1 (9.1%)	1 (9.1%)	0 (0%)	0.82
Control 9	5 (71.4%)	2 (28.6%)	0 (0%)	0 (0%)	0 (0%)	0.29
Control 10	6 (50%)	3 (25%)	1 (8.3%)	1 (8.3%)	1 (8.3%)	1.00
Control 11	4 (80%)	0 (0%)	1 (20%)	0 (0%)	0 (0%)	0.40
Control 12	12 (85.7%)	1 (7.14%)	1 (7.14%)	0 (0%)	0 (0%)	0.21
Control 13	4 (40%)	3 (30%)	1 (10%)	1 (10%)	1 (10%)	1.20
Control 14	8 (42.1%)	6 (31.6%)	1 (5.3%)	3 (15.8%)	1 (5.3%)	1.11
Control 15	10 (62.5%)	0 (0%)	2 (12.5%)	3 (18.75%)	1 (6.25%)	1.06
Control 16	7 (70%)	2 (20%)	1 (10%)	0 (0%)	0 (0%)	0.40
Control 17	4 (30.8%)	4 (30.8%)	0 (0%)	4 (30.8%)	1 (7.7%)	1.54
Average Percentage:	51.1%	23.1%	11.5%	9.7%	4.7%	Average Progression Score: 0.95

(B)

Individual Animals	Normal Number	PanIN1 Number	PanIN2 Number	PanIN3 Number	Carcinoma Number	Progression Score
Montelukast 0.1 1	1 (12.5%)	3 (37.5%)	1 (12.5%)	2 (25%)	1 (12.5%)	1.88
Montelukast 0.1 2	3 (75%)	0 (0%)	1 (25%)	0 (0%)	0 (0%)	0.50
Montelukast 0.1 3	1 (20%)	0 (0%)	2 (40%)	2 (40%)	0 (0%)	2.00
Montelukast 0.1 4	4 (44.4%)	0 (0%)	3 (33.3%)	1 (11.1%)	1 (11.1%)	1.44
Montelukast 0.1 5	6 (60%)	0 (0%)	1 (10%)	2 (20%)	1 (10%)	1.20
Montelukast 0.1 6	2 (22.2%)	2 (22.2%)	3 (33.3%)	2 (22.2%)	0 (0%)	1.56
Montelukast 0.1 7	6 (60%)	2 (20%)	2 (20%)	0 (0%)	0 (0%)	0.60
Montelukast 0.1 8	9 (75%)	2 (16.7%)	1 (8.3%)	0 (0%)	0 (0%)	0.33
Montelukast 0.1 9	6 (66.7%)	2 (22.2%)	1 (11.1%)	0 (0%)	0 (0%)	0.44
Montelukast 0.1 10	5 (62.5%)	0 (0%)	3 (37.5%)	0 (0%)	0 (0%)	0.75
Montelukast 0.1 11	11 (84.6%)	1 (7.7%)	0 (0%)	1 (7.7%)	0 (0%)	0.31

Montelukast 0.1$_{12}$	8 (72.7%)	0 (0%)	1 (9.1%)	2 (18.2%)	0 (0%)	0.73
Montelukast 0.1$_{13}$	8 (53.3%)	5 (33.3%)	1 (6.7%)	0 (0%)	1 (6.7%)	0.73
Montelukast 0.1$_{14}$	11 (73.3%)	1 (6.7%)	0 (0%)	2 (13.3%)	1 (6.7%)	0.73
Montelukast 0.1$_{15}$	5 (35.7%)	4 (28.6%)	2 (14.3%)	2 (14.3%)	1 (7.1%)	1.28
Average Percentage:	54.5%	13.0%	17.4%	11.5%	3.6%	Average Progression Score : 0.97

(C)

Individual Animals	Normal Number	PanIN1 Number	PanIN2 Number	PanIN3 Number	Carcinoma Number	Progression Score
Montelukast 0.2$_{1}$	6 (60%)	1 (10%)	1 (10%)	1 (10%)	1 (10%)	1.00
Montelukast 0.2$_{2}$	8 (61.5%)	2 (15.4%)	3 (23.1%)	0 (0%)	0 (0%)	0.62
Montelukast 0.2$_{3}$	9 (90%)	1 (10%)	0 (0%)	0 (0%)	0 (0%)	0.10
Montelukast 0.2$_{4}$	4 (57.1%)	1 (14.3%)	2 (28.6%)	0 (0%)	0 (0%)	0.71
Montelukast 0.2$_{5}$	5 (38.5%)	5 (38.5%)	0 (0%)	2 (15.4%)	1 (7.7%)	1.15
Montelukast 0.2$_{6}$	6 (66.7%)	0 (0%)	1 (11.1%)	1 (11.1%)	1 (11.1%)	1.00
Montelukast 0.2$_{7}$	7 (77.8%)	1 (11.1%)	1 (11.1%)	0 (0%)	0 (0%)	0.33
Montelukast 0.2$_{8}$	8 (80%)	0 (0%)	1 (10%)	1 (10%)	0 (0%)	0.50
Montelukast 0.2$_{9}$	7 (63.6%)	1 (9.1%)	3 (27.3%)	0 (0%)	0 (0%)	0.64
Montelukast 0.2$_{10}$	7 (50%)	3 (21.4%)	0 (0%)	2 (14.3%)	2 (14.3%)	1.21
Montelukast 0.2$_{11}$	7 (87.5%)	0 (0%)	0 (0%)	1 (12.5%)	0 (0%)	0.38
Montelukast 0.2$_{12}$	6 (75%)	0 (0%)	2 (25%)	0 (0%)	0 (0%)	0.50
Montelukast 0.2$_{13}$	10 (76.9%)	1 (7.7%)	2 (15.4%)	0 (0%)	0 (0%)	0.38
Montelukast 0.2$_{14}$	4 (80%)	0 (0%)	1 (20%)	0 (0%)	0 (0%)	0.40
Montelukast 0.2$_{15}$	8 (72.7%)	1 (9.1%)	1 (9.1%)	1 (9.1%)	0 (0%)	0.55
Average Percentage:	69.2% *	9.8% **	12.7%	5.5%	2.9%	Average Progression Score: 0.63

(D)

Individual Animals	Normal Number	PanIN1 Number	PanIN2 Number	PanIN3 Number	Carcinoma Number	Progression Score
Montelukast 0.4$_{1}$	7 (70%)	0 (0%)	3 (30%)	0 (0%)	0 (0%)	0.60
Montelukast 0.4$_{2}$	8 (80%)	1 (10%)	1 (10%)	0 (0%)	0 (0%)	0.30
Montelukast 0.4$_{3}$	2 (40%)	2 (40%)	1 (20%)	0 (0%)	0 (0%)	0.80
Montelukast 0.4$_{4}$	7 (77.8%)	1 (11.1%)	1 (11.1%)	0 (0%)	0 (0%)	0.33
Montelukast 0.4$_{5}$	7 (58.3%)	3 (25%)	1 (8.3%)	0 (0%)	1 (8.3%)	0.75
Montelukast 0.4$_{6}$	3 (60%)	1 (20%)	1 (20%)	0 (0%)	0 (0%)	0.60
Montelukast 0.4$_{7}$	13 (76.5%)	2 (11.8%)	0 (0%)	1 (5.9%)	1 (5.9%)	0.53
Montelukast 0.4$_{8}$	8 (57.1%)	3 (21.4%)	1 (7.1%)	1 (7.1%)	1 (7.1%)	0.86
Montelukast 0.4$_{9}$	5 (71.4%)	2 (28.6%)	0 (0%)	0 (0%)	0 (0%)	0.29
Montelukast 0.4$_{10}$	8 (88.9%)	1 (11.1%)	0 (0%)	0 (0%)	0 (0%)	0.11
Montelukast 0.4$_{11}$	4 (40%)	1 (10%)	2 (20%)	2 (20%)	1 (10%)	1.50
Montelukast 0.4$_{12}$	6 (60%)	3 (30%)	1 (10%)	0 (0%)	0 (0%)	0.50
Montelukast 0.4$_{13}$	7 (70%)	3 (30%)	0 (0%)	0 (0%)	0 (0%)	0.30
Montelukast 0.4$_{14}$	11 (84.6%)	1 (7.7%)	1 (7.7%)	0 (0%)	0 (0%)	0.23
Montelukast 0.4$_{15}$	11 (78.6%)	0 (0%)	1 (7.1%)	2 (14.3%)	0 (0%)	0.57
Average Percentage:	67.5% *	17.1%	10.1%	3.2%	2.1%	Average Progression Score: 0.55 *

*$p < 0.05$ and **$p < 0.01$ compared with controls.

Table S6. Patient's characteristics.

Parameters	CYSLTR1 Expression		*p* Value

	**High (n = 43)**	**Low (n = 65)**	
	**Gender**		
Male	29	45	0.845
Female	14	20	
	**Age**		
Median (range)	69 (32-85)	69 (45-84)	0.972
	**Differentiation**		
Well	20	23	0.193
Moderate	13	16	
Poorly	10	26	
	**Stage**		
IA, IB	3	5	0.984
IIA, IIB	38	57	
III	1	1	
IV	1	2	
	**pT Stage**		
pT1	2	5	0.87
pT2	3	3	
pT3	37	56	
pT4	1	1	
	**Lymph Node Metastasis**		
negative	14	27	0.347
positive	29	38	
	**Distant Organ Metastasis**		
negative	42	63	0.816
positive	1	2	

Table S7. Antibodies and dilutions for immunohistochemistry. Antigen retrieval of CYSLTR1 and Ki-67 were performed with heat activation in high, low and high pH buffer, respectively.

**Protein**	**Dilution**	**Antibodies**
CYSLTR1	100	Abcam plc. (Cambridge, UK)
Ki-67	100	Clone SP6, Thermo Fisher Scientific (Waltham, MA)
Phospho-ERK1/2	1000	Cell Signaling Technology, Inc. (Danvers, MA)

Table S8. Antibodies and dilutions for immunoblotting.

**Protein**	**Dilution**	**Antibodies**
β-Actin	5000	Abcam plc. (Cambridge, UK)
CYSLTR1	1000	Abcam plc. (Cambridge, UK)
ERK1/2	1000	Cell Signaling Technology, Inc. (Danvers, MA)
Phospho-ERK1/2	1000	Cell Signaling Technology, Inc. (Danvers, MA)
PARP	1000	Cell Signaling Technology, Inc. (Danvers, MA)
